# Assessing the cost-effectiveness of capnography for end-tidal CO_2_ monitoring during in-hospital cardiac arrest: A middle-income country perspective analysis

**DOI:** 10.1016/j.ahjo.2024.100373

**Published:** 2024-02-29

**Authors:** Sérgio Renato da Rosa Decker, Lucas Emanuel Marzzani, Pedro Rotta de Ferreira, Paulo Ricardo Mottin Rosa, Janete Salles Brauner, Regis Goulart Rosa, Eduardo Gehling Bertoldi

**Affiliations:** a Programa de Pós-graduação em Cardiologia e Ciências Cardiovasculares, Universidade Federal do Rio Grande do Sul, Porto Alegre, Brazil; b Serviço de Medicina Interna, Hospital Moinhos de Vento, Porto Alegre, Brazil; c Hospital de Clínicas de Porto Alegre, Porto Alegre, Brazil; d Instituto de Cardiologia – Fundação Universitária de Cardiologia, Porto Alegre, Brazil; e Departamento de Medicina Interna, Hospital Nossa Senhora da Conceição, Porto Alegre, Brazil; f Faculdade de Medicina, Universidade Federal de Pelotas, Pelotas, Brazil

**Keywords:** Cost-effectiveness, End-tidal carbon dioxide, Capnography, In-hospital, Cardiac arrest, Advanced cardiac life support

## Abstract

**Study objective:**

To evaluate the cost-effectiveness of EtCO_2_ monitoring during in-hospital cardiorespiratory arrest (CA) care outside the intensive care unit (ICU) and emergency room department.

**Design:**

We performed a cost-effectiveness analysis based on a simple decision model cost analysis and reported the study using the CHEERS checklist. Model inputs were derived from a retrospective Brazilian cohort study, complemented by information obtained through a literature review. Cost inputs were gathered from both literature sources and contacts with hospital suppliers.

**Setting:**

The analysis was carried out from the perspective of a tertiary referral hospital in a middle-income country.

**Participants:**

The study population comprised individuals experiencing in-hospital CA who received cardiopulmonary resuscitation (CPR) by rapid response team (RRT) in a hospital ward, not in the ICU or emergency room department.

**Interventions:**

Two strategies were assumed for comparison: one with an RRT delivering care without capnography during CPR and the other guiding CPR according to the EtCO_2_ waveform.

**Main outcome measures:**

Incremental cost-effectiveness rate (ICER) to return of spontaneous circulation (ROSC), hospital discharge, and hospital discharge with good neurological outcomes.

**Results:**

The ICER for EtCO_2_ monitoring during CPR, resulting in an absolute increase of one more case with ROSC, hospital discharge, and hospital discharge with good neurological outcome, was calculated at Int$ 515.78 (361.57–1201.12), Int$ 165.74 (119.29–248.4), and Int$ 240.55, respectively.

**Conclusion:**

In managing in-hospital CA in the hospital ward, incorporating EtCO2 monitoring is likely a cost-effective measure within the context of a middle-income country hospital with an RRT.

## Highlights

• Cost-effectiveness analysis of EtCO2 monitoring during in-hospital cardiorespiratory arrest management.
• EtCO2 monitoring has demonstrated a potential to improve outcomes in cardiopulmonary resuscitation (CPR).
• We use a simple decision tree model to assess the cost per ROSC, hospital discharge, and good neurological outcomes.
• The incremental cost-effectiveness rate using EtCO2 monitoring was well below willingness-to-pay thresholds worldwide.
• EtCO2 monitoring during CPR probably is a cost-effective measure for health systems, even in low- and middle-income countries.

## 1 Introduction

Despite recent improvements in outcomes after cardiorespiratory arrest (CA) [1], mortality remains high. The incidence of CA events ranges from 1 to 10 per 1000 hospital admissions worldwide [2]. Hospital discharge rates for in-hospital CA range from 0 % to 42 %; among those discharged, up to 66.3 % to 85 % have favorable neurological outcomes [[2], [3], [4]]. In addition to the direct impact on the health of individuals, CA generates high costs, and 17 % of this amount is due to post-CA hospitalizations [5,6].

The 2020 International Liaison Committee (ILCOR) guidelines cited monitoring end-tidal CO_2_ (EtCO_2_) with continuous waveform capnography during cardiopulmonary resuscitation (CPR) as a reasonable option to measure the quality of CPR maneuvers [[7], [8], [9]]. These recommendations were based on studies that showed an increased return of spontaneous circulation (ROSC), hospital discharge, and potentially more discharges with favorable neurological outcomes using EtCO_2_ monitoring or other CPR feedback quality measures [[7], [8], [9], [10]]. These CPR quality measures are commonly used in intensive care units (ICUs) or emergency room departments [9]; however, implementing these technologies in hospital wards demands a better understanding of cost-effectiveness.

Only a few studies evaluated EtCO_2_ monitoring cost-effectiveness [12]; to our knowledge, none refer to CA. Therefore, the present study aimed to evaluate the cost-effectiveness of EtCO_2_ monitoring during in-hospital CA, specifically their use by rapid response teams (RRT) in the hospital ward, by calculating the incremental cost-effectiveness rate (ICER) to ROSC, hospital discharge, and hospital discharge with good neurological outcomes. The cost-effectiveness analysis was performed from the perspective of a tertiary referral hospital in the Brazilian Unified Health System (SUS), a middle-income country.

## 2 Methods

### 2.1 Analysis plan and model design

We performed a cost-effectiveness analysis based on a simple decision model according to current recommendations and reported the study according to the Consolidated Health Economic Evaluation Reporting Standards 2022 (CHEERS 2022) checklist [[13], [14], [15]]. The Hospital Nossa Senhora da Conceição (Brazil) ethical committee approved our plan analysis (approval number 5.896.920).

The decision to employ a simple decision tree design for the model was driven by its inherent simplicity in capturing the research problem and the absence of interpersonal interactions in our model [15]. The rationale behind opting for simpler models extends from their ease of comprehension to their consequent ease of validation, setting them apart from more intricate models such as Markov models [15]. As our analysis primarily concentrates on the short-term, specifically focusing on the period from hospitalization to discharge at most, the probability inputs for transitioning between states of illness and health are not anticipated to fluctuate within this timeframe. Hence, complex models are not deemed necessary to address these variations [15].

### 2.2 Study population

Our study population was based on a retrospective cohort record of the internal medicine service at Hospital Nossa Senhora da Conceição, a tertiary referral teaching hospital associated with the SUS, localized in Porto Alegre, Brazil, and baseline characteristics of the patients in the studies from which we took the efficacy inputs for our decision model [8,9]. This retrospective cohort was referred to four years before the COVID-19 pandemic, was approved by the same ethical committee (approval number 3.539.905), and was made using an Utstein-style registry. The baseline characteristics of our retrospective cohort are presented in Table 1.

**Table 1:** In-hospital cardiorespiratory arrest characteristics from a retrospective cohort at tertiary Hospital Nossa Senhora da Conceição (Porto Alegre, Rio Grande do Sul, Brazil), using an Utstein-style registry, referring to a period of 4 years prior to the COVID-19 pandemic.

	n (%) 364 (100)
Female	165 (45.3)
Age (mean [SD])	64.9 ± 14.8
Basic life support at RRT arrival	296 (81.3)
**Initial rhythm**	
PEA	224 (61.5)
Asystole	92 (25.3)
Ventricular fibrillation	42 (11.5)
Ventricular tachycardia	6 (1.6)
Defibrillation delivery	99 (27.2)
**Drugs used**	
Adrenaline	349 (95.9)
Sodium bicarbonate	144 (39.6)
Amiodarone	53 (14.6)
Sodium gluconate	51 (14.0)
**Source of the CA**	
Non-cardiac	312 (85.7)
Cardiac	45 (12.4)
Undetermined	7 (1.9)
**Presumed CA etiology**	
Respiratory failure	213 (58.5)
Acute coronary syndrome	41 (11.3)
Metabolic	35 (9.6)
Hypovolemia	18 (4.9)
Pulmonary thromboembolism	12 (3.3)
Neurologic	4 (1.1)
Arrhythmia	3 (0.8)
Cardiac tamponade	2 (0.5)
Undetermined	7 (1.9)
Advanced stage neoplasm	62 (17)
Sepsis diagnosis before CA	206 (56.6)
**Cohort outcomes**	
ROSC	144 (39.6)
Hospital discharge	35 (9.6)
Hospital discharge with CPC 1 or 2	24 (6.6)

95 % CI - 95 % confidence interval; SD - Standard deviation; RRT – Rapid response team; PEA – pulseless electrical activity; CA – cardiorespiratory arrest; ROSC – return of spontaneous circulation; CPC - Cerebral Performance Category.

Thus, the case mix with pooled results from our cohort and literature used for our model included hospitalized adults about fifty to seventy years old (mean 64.44 years), an equal gender mix (45.3–60.7 % female), and a high percentage of sepsis with respiratory insufficiency as the etiology of in-hospital worsening leading do CA (Table 1; references [8, 9]). Our retrospective base cohort includes just CA in the hospital ward and did not include CA that occurred in the emergency room department or ICU.

### 2.3 Study intervention

Cost-effectiveness analysis was conducted from the perspective of a tertiary hospital associated with the SUS. Two strategies were assumed for comparison: one with a RRT delivering care for patients in the hospital ward without capnography during CPR and the other guiding CPR according to the EtCO_2_ waveform, seeking to optimize CPR in cases of EtCO_2_ < 10 mmHg [7,9].

### 2.4 Model and inputs

**Fig. 1:**
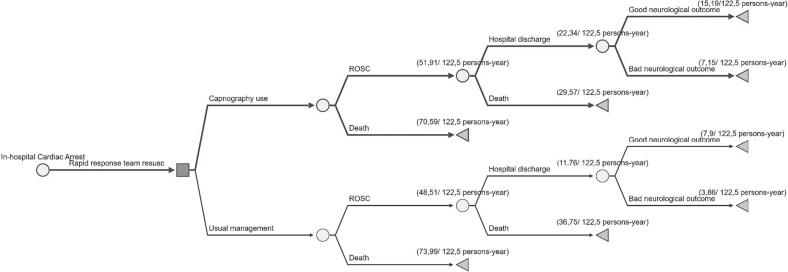
Decision tree for cost-effectiveness of EtCO2 monitoring with capnography by rapid response team. We assume 25,000 hospitalizations and a total of 122.5 in-hospital cardiorespiratory arrest per year, according with our case-base cohort from a tertiary hospital at a middle-income country (Brazil).

We built a decision tree (Fig. 1) using the software SilverDecisions (version 1.1.0, 2021) with a time horizon of one year. In the cohort-based case, the RRT serves all hospital wards except the ICU and emergency room. Cost-effectiveness was expressed in ICER per ROSC, per hospital discharge, and per discharge with good neurological outcome (a Cerebral Performance Category of 1 or 2).

We considered 25,000 admissions per year, as our hospital website shows (https://www.ghc.com.br/default.asp?idMenu=unidades&idSubMenu=1, accessed March 31, 2023), and according to a trend observed (Table 2). Our case-based cohort showed approximately 4.9 CA in the hospital ward per 1000 admissions (Table 2); 39.6 % ROSC, 9.6 % were discharged from the hospital, and 68.6 % of discharges had a good neurological outcome (Table 1). Thus, we estimate 122.5 CA events per year managed by RRT and a baseline incidence (without the use of capnography to guide CPR) of 1.9, 0.47, and 0.32 per 1000 hospitalized patients to achieve ROSC, hospital discharge, and discharge with good neurologic outcomes, respectively (Table 1, Table 2; Fig. 1).

**Table 2:** Cardiorespiratory arrest per year and admissions at tertiary hospital Hospital Nossa Senhora da Conceição (Porto Alegre, Rio Grande do Sul, Brazil), referring to four years before the COVID-19 pandemic.

Year	CA per year	Admissions	Admissions/1000	CA/1000 admissions per year
2015	81	16,393	16.39	4.9
2016	108	18,624	18.62	5.8
2017	94	19,651	19.65	4.8
2018	81	20,310	20.31	4.0
Total	364	74,978	74.98	4.9

CA – cardiorespiratory arrest.

Efficacy probability in achieving ROSC, hospital discharge, and discharge with good neurologic outcome using EtCO_2_ monitoring during CPR was extracted [[7], [8], [9]] primarily from the articles cited by the most recent ILCOR guidelines in the section that discusses recommendations for CPR feedback and monitoring [7]. The model inputs were selected according to the sequence of events in our probabilistic tree (ROSC, hospital discharge, and discharge with good neurologic outcome) and were selected, prioritizing randomized clinical trials as a source. In the case of a lack of randomized clinical trials addressing the impact of the technology on desired outcomes, we choose observational studies with adjustment for propensity score or instrumental variables that evaluated the use of EtCO_2_ monitoring as an intervention. As a third resource, we searched randomized clinical trials that assess CPR feedback quality measurement, like EtCO_2_ monitoring, during CA as an intervention and if there is a benefit for the intended outcome. We show this predefined order to select the level of evidence for efficacy inputs in Supplementary Appendix Table 1.

We did not find any clinical trial addressing EtCO2 monitoring as an intervention in CA. Thus, for the ROSC outcome, we use an observational study with a large sample size and propensity score adjustment that tested EtCO_2_ monitoring as an intervention with a risk ratio (RR) for ROSC of 1.07 (95 % confidence interval [CI] 1.03–1.1) [9].

Considering an EtCO_2_ > 10 mmHg versus <10 mmHg, the same study showed a significant increase in patients discharged from the hospital with good neurological outcomes (RR 11.3, 95 % CI 7.6–16.7, p 0.004) [9]. Nonetheless, we adopted a more conservative probability for this outcome and used a fixed probability rate input for discharge with a good neurological outcome because deciding to monitor EtCO_2_ during CPR does not guarantee achieving >10 mmHg in all cases [9]. Thus, according to our baseline probability, we assume a 68.6 % incidence of good neurological outcomes from hospital discharge patients in both decision tree arms (Table 1).

For hospital discharge outcomes, we extract the input probabilities from a randomized clinical trial that tested quality feedback mechanisms for CPR as an intervention with an RR of 1.9 (95 % CI 1.6–2.25) [8]. Table 3 summarizes the decision model inputs. Finally, for discussion purposes, we showed the pooled results with studies selected for efficacy inputs; however, due to differences in study design, we chose not to use the pooled results for model inputs.

**Table 3:** Model inputs.

Variable	Base-case	References	Sensitivity analysis (95 % CI)	References
*Probabilities*				
ROSC control (incidence/year^a^)	48.51	c	–	–
ROSC EtCO2 (incidence/year^a^)	51.91	[9]	49.97–53.36	[9]
Hospital discharge control (incidence/year^a^)	11.76	c	–	–
Hospital discharge EtCO2 (incidence/year^a^)	22.34	[8]	18.82–26.46	[8]
Good neurologic outcome^b^ control (incidence/year^a^)	7.9	c	–	–
Good neurologic outcome^b^ EtCO2 (incidence/year^a^)	15.19	d	–	–


Variable	Costs (Int$)	References	Sensitivity analysis (Int$)	References
Monitor cost (unit)	5250.94	[12]	4140.7–7902.23	[12]
Cannula cost (total/year)	659.29	[12]^c^	–	–
Total additional cost per EtCO2 monitoring (total budget cost^e^/total events per year^a^)	1753.64	[12]^c^	1369.78–2106.46	[12]^c^

95 % CI – 95 % Confidence Interval; ROSC - Return of spontaneous circulation; EtCO2 - End-tidal CO2 monitoring; Int$ - International dollar.

a We assume 25,000 hospitalizations and a total of 122.5 cardiorespiratory arrests per year.

b Good neurologic outcome according to Cerebral Performance Category.

c Our retrospective cohort according to Utstein registry (Table 1, Table 2).

d Assumption.

e We assume a necessity of one monitor per hospital ward.

Because the EtCO_2_ monitor is a reusable technology, the costs were divided by the total estimated CA attended. The monitoring costs and line to capnography measures were extracted from another cost-effectiveness study addressing the EtCO2 monitoring outside the CA setting, evaluating their use for orotracheal intubation aiming to decrease catastrophic events, and considering the two devices' costs (monitor and line to capnography measures) [12]; we have compared these costs with our suppliers' providers through email and call contact to understand if there are some critical differences in costs. Inputs regarding baseline rate, intervention efficacy, and costs are presented in Table 3. We consider a single EtCO_2_ monitor taken by the RRT to each CA, not an EtCO_2_ monitor integrated with defibrillators. We did not consider making our model other applications for EtCO_2_, for example, evaluating the correct placement of a tracheal tube.

### 2.5 Currency, conversion, and discount rate

We presented the costs in international dollars (Int$) to extrapolate and understand the results from an international perspective, considering the conversion rate of Brazilian *real* to Int$ using the World Bank's latest available purchasing power parity conversion factor of 2.53 (https://data.worldbank.org/indicator/PA.NUS.PPP?locations=BR, accessed April 18, 2023). We did not calculate a discount rate due to a short time horizon in our model. The Int$, when compared to the US dollar, should be interpreted as a benchmark for assessing the purchasing power and exchange rate stability across countries. It represents a standardized unit of currency that eliminates the effects of exchange rate fluctuations and regional price differences, allowing for more accurate international comparisons; thus, researchers and policymakers can analyze economic indicators, providing valuable insights into global economic trends and disparities.

### 2.6 Sensitivity analysis and budget impact analysis

We analyzed sensitivity using the EtCO_2_ monitoring device's estimated cost and efficacy variability; this range varies more or less Int$ 3761.19 according to the literature [12] and according to the 95 % CI range of the efficacy studies (for ROSC and hospital discharge outcomes) [8,9] (Table 1).

Finally, we performed a budget impact analysis, which integrates cost information with epidemiological estimates from the perspective of a middle-income country hospital manager. We considered the total amount with EtCO_2_ monitors and additional expenses with increased hospitalization time due to increased ROSC rate. According to our registry (Table 1), we assumed that 58.5 % of CA occurred due to respiratory failure, and 56.6 % of patients were septic before the event. Therefore, we chose to use data from a macro-costing where the in-hospital cost of sepsis was evaluated in Brazil [17,18].

## 3 Results

The ICERs of using EtCO_2_ waveform monitoring during CPR by RRT in the hospital ward for an absolute increase of one more case with ROSC, hospital discharge, and hospital discharge with a good neurological outcome were Int$ 515.78, Int$ 165.74, and Int$ 240.55, respectively (Table 4).

**Table 4:** Incremental cost-effectiveness range (ICER) according to base-case inputs and sensitivity analysis.

Results per outcome	ICER	ICER sensitivity analysis according to the efficacy	ICER sensitivity analysis according to costs
ROSC (Int$)	515.78	361.57–1201.12	402.88–628.66
Hospital discharge (Int$)	165.74	119.29–248.4	129.48–202.03
Good neurologic outcome^a^ (Int$)	240.55	–	–

ROSC - Return of spontaneous circulation; Int$ - Internationl dollar.

a Cerebral Performance Category 1 or 2.

In the sensitivity analysis, we observed that the ICER ranged from Int$ 361.57 to 1201.12 per ROSC and Int$ 119.29 to 248.4 for hospital discharge, depending on the anticipated efficacy, which varied in accordance with the 95 % CI of the RR from the studies utilized for efficacy inputs. In the same sense, the ICER varied between Int$ 402.88 to 628.66 per ROSC, and Int$ 129.48 to 202.03 per hospital discharge, reflecting the expected variation of costs values with EtCO_2_ monitoring equipment (Table 4).

From the perspective of our tertiary referral hospital and the budgetary impact over one year, we estimate that there would be an increase of Int$ 5892.88 (for the period of stay in the ICU) and Int$ 1220.89 (for the period of stay in the hospital ward) for each additional patient who reached ROSC using EtCO_2_ monitoring plus the total expenditure with the purchase of monitors and cannula to EtCO_2_ monitoring. Therefore, from a one-year perspective, an additional Int$ 24,181.16 plus Int$ 134,717.67 would be spent (total budget impact of Int$ 158,898.83).

## 4 Discussion

Our findings suggest that using EtCO_2_ monitoring during the CA care by the RRT in a hospital ward from a tertiary referral hospital associated with the SUS is cost-effective for ROSC outcomes, hospital discharge, and hospital discharge with good neurological outcomes. Many countries have sought to standardize a value to guide decisions regarding incorporating new technologies into healthcare systems by establishing a cost-effectiveness threshold, which the acceptable ICER represents for a determinate outcome (willingness-to-pay [WTP] threshold). ICER is a ratio with the monetary cost of the intervention in the numerator and his health outcomes in the denominator. Thus, it is essential to understand the effectiveness of the proposed therapy, costs, and the WTP threshold from the perspective of the health system or stakeholder. Currently, the WTP threshold recommended by the Brazilian government for the quality-adjusted life year outcome is Int$ 15,810; worldwide, the mean WTP for the same outcome is Int$ 34,309 [19,20]. The values found in our analysis are well below these thresholds. However, our work did not evaluate quality-adjusted life years; therefore, we followed other studies that used the WTP threshold reference for other relevant outcomes [21,22].

In our sensitivity analyses, EtCO_2_ monitoring during the CA remained cost-effective even in higher costs and worse therapeutic efficacy scenarios. Because the costs of monitors and the EtCO_2_ line cannula may be even lower than we found, considering the popularity of the technology, improved forms of purchase for most prominent institutions, or considering EtCO_2_ monitoring integrated with defibrillators, the results are even more promising. Furthermore, in the budget impact analysis for our middle-income country perspective, despite an increase in the total amount spent on hospital admissions due to the increase in patients who achieve ROSC (Int$ 24,181.16), the hospital and health system failed to spend (in relative terms) almost twice the amount with hospitalizations after ROSC that would not reach hospital discharge without the use of technology (Int$ 51,076.82).

Our study has some limitations. Regarding the intervention and the extent to which its benefit is established, there are few studies assessing the effectiveness of interventions during CA, even for well-established therapies, and only about 10 % of recommendations are based on randomized clinical trials; thus, ILCOR guidelines proposed EtCO_2_ monitoring during CA as a weak recommendation due to limited data [23]. However, two essential papers evaluating the use of physiological monitoring and the use of feedback devices during CPR to optimize care supported our findings [8,9]. Evaluating the pooled efficacy of these studies, using RR as an effect measure, we found an RR of 1.09 (95 % CI 1.06–1.12; *p* < 0.001; number needed to treat 18) and an RR of 1.25 (95 % CI 1.15–1.36; p < 0.001; number needed to treat 22) for ROSC and hospital discharge outcomes, respectively. The latter remains cost-effective regarding the worst scenario possible, with an ICER of Int$ 994.13 per additional patient who achieves hospital discharge with intervention. Concerning the quality of the cost data, better accuracy could be provided by a more detailed micro-costing analysis, which provides a greater understanding of the cost of caring for complex patients, but it was not feasible [24].

Another concern and limitation of our model regarding efficacy inputs is the intricate relationship between EtCO_2_ values and CA etiology. Our baseline cohort population has respiratory failure as a primary mechanism of CA, and studies have shown high EtCO_2_ values at the beginning of CPR maneuvers in these cases, which can work as an essential confounder of the capnography monitoring [25,26]. However, these initial high values typically decrease after some resuscitation breaths, displaying an EtCO_2_ “U” shape from the CA to the ROSC (high values, low values, and high values) [[25], [26], [27]]. While these fluctuations may minimally interfere with decision-making processes by RRT, our model did not incorporate this aspect. Moreover, our studies for efficacy probabilities have considered this phenomenon since they evaluate in-hospital CA patients with respiratory failure and pulseless electrical activity as primary CA etiology and rhythm [8,9].The generalizability of our findings holds significant implications for other tertiary hospitals in middle and low-income countries, where the demand for ICU beds in health systems exceeds supply due to the limited number of hospitals equipped to handle complex cases [28]. Consequently, as a means of enhancing the resilience of health systems, there is a notable frequency of RRT activations to provide support in hospital wards and triage the patients, particularly during high-strain periods of the healthcare systems, as we could see during the pandemic period [29,30].

In conclusion, we advocate for adopting capnography as a technology for monitoring the quality of CPR, owing to its affordability and potential to significantly influence outcomes even when we evaluate their use in non-monitored units. Nonetheless, further clinical trials incorporating nested cost evaluations are imperative to enhance our comprehension of these capabilities and to mitigate the inherent uncertainties in our research design [31], where we make several assumptions, thus warranting cautious extrapolation of results.

The following is the supplementary data related to this article.

Supplementary Appendix Table 1 Hierarchical Selection Order of Efficacy Inputs Based on Level of Evidence. Supplementary Appendix Table 1

## CRediT authorship contribution statement

**Sérgio Renato da Rosa Decker:** Writing – review & editing, Writing – original draft, Supervision, Software, Resources, Project administration, Methodology, Investigation, Funding acquisition, Formal analysis, Data curation, Conceptualization. **Lucas Emanuel Marzzani:** Writing – original draft, Project administration, Conceptualization. **Pedro Rotta de Ferreira:** Writing – original draft, Data curation, Conceptualization. **Paulo Ricardo Mottin Rosa:** Writing – original draft, Supervision. **Janete Salles Brauner:** Writing – review & editing, Methodology, Formal analysis, Data curation, Conceptualization. **Regis Goulart Rosa:** Writing – review & editing. **Eduardo Gehling Bertoldi:** Writing – review & editing, Supervision, Software, Methodology, Formal analysis, Data curation, Conceptualization.

## Declaration of competing interest

The authors declare the following financial interests/personal relationships which may be considered as potential competing interests: Sergio Renato da Rosa Decker reports financial support was provided by 10.13039/501100002322Coordenação de Aperfeiçoamento de Pessoal de Nível Superior (CAPES). The authors of this article declare that they have no other conflicts of interest to disclose.
