# Supplementary material for: Assessing the cost-effectiveness of capnography for end-tidal CO2 monitoring during in-hospital cardiac arrest: A middle-income country perspective analysis
Source: Am Heart J Plus. 2024 Feb 29;40:100373. doi: 10.1016/j.ahjo.2024.100373 (PMC10946019; doi:10.1016/j.ahjo.2024.100373)
Supplement: Supplementary Appendix Table 1 — Hierarchical Selection Order of Efficacy Inputs Based on Level of Evidence. [file mmc1.docx]

**Author contributions**

Sérgio Renato da Rosa Decker

Conceptualization; Data curation; Formal analysis; Funding acquisition; Investigation; Methodology; Project administration; Resources; Software; Supervision; Roles/Writing - original draft; Writing - review & editing.

Lucas Emanuel Marzzani

Conceptualization; Project administration; Roles/Writing - original draft;

Pedro Rotta de Ferreira

Conceptualization; Data curation; Roles/Writing - original draft;

Paulo Ricardo Mottin Rosa

Supervision; Writing - review & editing.

Janete Salles Brauner

Conceptualization; Data curation; Formal analysis; Methodology; Writing - review & editing.

Regis Goulart Rosa

Writing - review & editing.

Eduardo Gehling Bertoldi

Conceptualization; Data curation; Formal analysis; Methodology; Software; Supervision; Writing - review & editing.

Supplementary Appendix Table 1: Hierarchical Selection Order of Efficacy Inputs Based on Level of Evidence.

| Order | Study design |
| --- | --- |
| 1st | Randomized clinical trial addressing EtCO2 monitoring as intervention during CA care |
| 2nd | Observational studies with adjustment for propensity score or instrumental variables that evaluated the use of EtCO_2_ monitoring as an intervention during CA care |
| 3rd | Randomized clinical trial that assess any CPR feedback quality measurement, like EtCO2 monitoring, as an intervention during CA care |
| 4th | Assumptions according to baseline probabilities observed in our base-case cohort |

Legend: EtCO2 – end-tidal CO_2;_ CA – cardiac arrest; CPR - cardiopulmonary resuscitation.
